# Si_3_N_4_ Microring Resonator-Based Refractive Index Sensing for Liquid Samples: Comparing Wavelength Scanning and Fixed-Wavelength Probing

**DOI:** 10.1021/acsmeasuresciau.5c00139

**Published:** 2025-12-15

**Authors:** Daniela Tomasetig, Jesus Hernan Mendoza-Castro, Silvia Schobesberger, Artem S. Vorobev, Liam O’Faolain, Bernhard Lendl

**Affiliations:** † TU Wien, 27259 Institute of Chemical Technologies and Analytics, Getreidemarkt 9/164, Vienna 1060, Austria; ‡ TU Wien, Institute of Applied Synthetic Chemistry, Getreidemarkt 9/163, Vienna 1060, Austria; § Centre for Advanced Photonics & Process Analysis, 587895 Munster Technological University, Rossa Avenue, Cork T12 P928, Ireland

**Keywords:** microring resonator, refractive index sensing, microfluidics, on-chip sensing, sugars

## Abstract

Measuring refractive index (RI) changes of liquid samples is central to many sensing applications including flow injection analysis, liquid chromatography, biosensing and photothermal spectroscopy. Commercial refractive index detectors optimized for liquid chromatography suffer from a limited linear range and measurement rate, restricting their use largely to separation sciences. In contrast, microring resonators (MRR) integrated with low-volume microfluidics, offer enhanced performance by minimizing sample dilution during flow-through RI measurements and increased dynamic range. MRRs realized by modern photonic integrated circuitry (PIC) technology also have the potential to be used as transducers in more advanced sensing schemes. Here, we demonstrate a silicon nitride (Si_3_N_4_) MRR integrated into a low-volume microfluidic system as a compact, chip-scale RI detector capable of real-time operation under dynamic flow conditions. Two interrogation modalities were experimentally compared for flow-through liquid sensing using the same MRR for the first time: resonance wavelength scanning for wide-range refractive index detection, and fixed-wavelength probing on the resonance slope for high-speed measurements. Using glucose solutions as test samples, the device was benchmarked against a commercial RI detector, achieving a sensitivity of 113 nm/RIU and a sLOD of 2.3 × 10^–6^ RIU (0.014 g/L glucose). To demonstrate the applicability of the developed RI-sensor for resolving transient RI peaks in realistic chromatographic flow conditions we also report its successful use in an isocratic separation of four sugars (sorbitol, fructose, glucose, and sucrose). These results highlight the potential of integrated Si_3_N_4_ MRRs as versatile, miniaturized transducers for quantitative, high-speed RI sensing in flow-based analytical systems.

## Graphical abstract

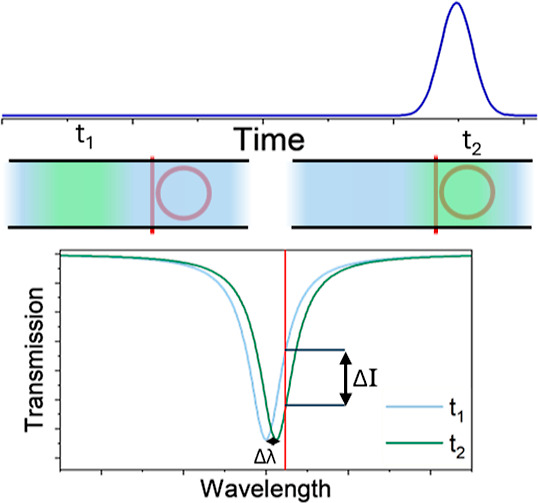

## Introduction

Accurate and time-resolved measurement of the refractive index (RI) of liquids is critical in applications where the composition of a flowing sample can change rapidly, such as flow injection analysis, liquid chromatography, reaction monitoring, and advanced sensing modalities such as photothermal spectroscopy. Traditional commercial RI detectors are limited by relatively slow measurement rates and large internal volumes, making them unsuitable for capturing subsecond variations in sample composition. Large measurement volumes generally lead to peak broadening and signal distortion, particularly in high-speed microfluidic systems.

Optical microring resonators (MRRs) integrated with microfluidics offer a powerful alternative for real-time RI measurements in rapidly changing flows.^1,2^ MRRs in photonic integrated circuitries (PICs) have been explored in the past for a range of applications including optical filtering, amplification, switching and sensing.^3–5^ MRRs enable sensing by exhibiting shifts in their resonance wavelength in response to changes in the refractive index of the surrounding medium. By application of a (bio)­chemically functionalized layer on top of a ring resonator, the (bio)­chemical selectivity of their response can be tuned,^6^ with the sensitivity of the underlying ring resonator as refractive index transducer still playing a central role in the performance of the overall sensing system. MRRs belong to the broader class of whispering gallery mode resonators,^7^ which also includes disk,^8^ spherical^9^ or toroidal resonators.^10^ MRRs have major advantages compared to other types of PIC devices capable of sensing refractive index changes, such as Mach–Zehnder interferometers^11,12^ or bimodal waveguides.^13^ While Mach–Zehnder Interferometers and bimodal waveguides can achieve similar performances,^14,15^ often cited advantages of MRR are their small footprint, easy fabrication and the fact that their resonance wavelength shifts linearly with refractive index changes.^16^ The MRR transduces RI changes as shifts in resonance wavelength and can be configured for high-speed operation with detection rates exceeding hundreds of Hertz. Due to their extremely small detection volumes and compatibility with low-dispersion microfluidic channels, MRRs minimize sample dilution and peak broadening, enabling precise tracking of fast, transient RI signals. This is especially important for applications where a fast transduction of the refractive index change is needed. Examples include high-speed separation processes, with peak widths in the order of a few seconds, photothermal spectroscopy, where refractive index changes need to be transduced within milli- to micro-seconds or the determination of binding kinetics and reaction kinetics with changes within subseconds to a few seconds.^17–20^

This work focuses on Si_3_N_4_ MRRs on an SiO_2_/Si platform and possible readout concepts for sensing RI changes. For incorporation in a microfluidic flow-through cell, sensors with a small footprint, such as MRRs, are needed. The choice of a Si_3_N_4_ on SiO_2_/Si platform was made due to its various advantages including compatibility with the complementary metal–oxide-semiconductor (CMOS) technology, low fabrication cost and higher tolerance to dimensional variations.^21,22^ As compared to a silicon-on-insulator (SOI) platform, Si_3_N_4_ offers lower waveguide losses, fewer spurious reflections and is less susceptible to thermal fluctuations, due to its lower thermo-optic coefficient of 2.45 × 10^–5^ K^–1^, compared to silicon with 1.87 × 10^–4^ K^–1^.^23–26^ In concurrent work, we recently established guidelines for the design and fabrication of MRR on this material platform.^27^

In most previous works, the refractive index change of the sample was detected by measuring the spectrum of the MRR with a tunable laser source^28,29^ or by using a broadband light source coupled to a spectrometer.^30^ While these read-out schemes offer high linearity, their measurement rate is inherently limited by the time needed to acquire the MRR spectrum. For applications, where refractive index changes need to be tracked rapidly, this is unacceptable. An alternative approach to track the refractive index change is to fix the laser wavelength to the steepest point of one resonance (i.e., the inflection point) and to record intensity changes (Δ*I*) at the MRR output caused by the shift of the resonance (Δλ), as shown in Figure 1. By additionally modulating the laser intensity and demodulating the recorded signal with a lock-in amplifier, the sensitivity can be further improved through noise suppression. In contrast commercial deflection-based refractive index detectors used in HPLC, are usually limited to a measurement range of 5 × 10^–4^ RIU measuring at 1–50 Hz.

**Figure 1:**
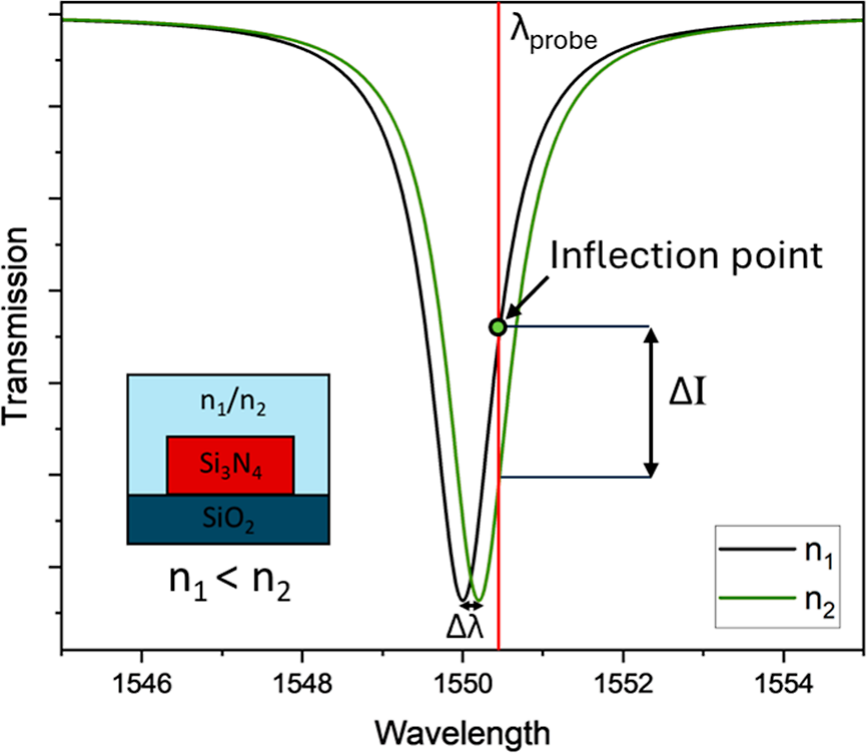
Schematic showing the ring resonator resonance response due to refractive index change of the media on top. The wavelength shift (Δλ) produces a variation in intensity (Δ*I*).

While similar read-out schemes have already been demonstrated in literature,^31,32^ we are, to the best of our knowledge, the first to experimentally compare both interrogation modalities under realistic flow conditions using the same MRR and to benchmark them against a commercial refractive index detector designed for liquid chromatography. Furthermore, to demonstrate the analytical relevance of the developed platform, we integrate the Si_3_N_4_ MRR detector into a HPLC setup and successfully resolve the separation of four sugars (sorbitol, fructose, glucose, and sucrose) using the fixed-wavelength interrogation modality, which has also not been done before, thus validating its performance in a practical chromatographic measurement scenario.

### Fundamentals of Ring Resonators for Refractive Index Sensing

In ring resonators, light is coupled from a straight bus waveguide into a second waveguide that is looped back on itself. When the optical path length of the ring is equal to an integer multiple of the wavelength of light coupled into the PIC, resonance takes place, leading to a drop in intensity measured at the output of the through (bus) waveguide. The dependence of the resonance wavelength (λ_res_) on the effective refractive index of the mode in the looped waveguide (*n*_eff_), the length of the looped waveguide (*L*) and the resonance order (*m*) can be described according to eq 1 ^33^

$$\lambda_{res}=\frac{L\times n_{eff}}{m}$$ 1

Different metrics have been introduced in the past to compare the performance of ring resonators.^34^ One important parameter is the bulk sensitivity, describing the change of the resonance wavelength (Δλ_res_) with the refractive index change in the cladding of the waveguide (Δ*n*_clad_)­

2 $$S=\frac{{\Delta \lambda }_{res}}{\Delta n_{clad}}$$

The bulk sensitivity can be determined experimentally by measuring the wavelength shift for multiple samples with a known refractive index (*S*_exp_). Additionally, it can be calculated using eq 3 with *n*_g_ being the group index of the resonator^35^

$$S_{theo}=\frac{1}{n_{g}}\frac{{\partial n}_{eff}}{\partial n_{clad}}\times \lambda_{res}$$ 3

Another important parameter for the characterization of resonances is the *Q*-factor. It can be approximated from the position of the resonance dip (ω_0_) and the full width at half-maximum (Δω). Generally, higher *Q*-factors improve the performance of ring resonators in refractive index sensing applications.^36–38^ However, previous studies have shown that good performances can also be achieved with moderate *Q*-factors,^39^ as other parameters, such as the resonance amplitude play an important role as well.^40^

4 $$Q=\frac{\omega_{0}}{\Delta \omega }$$

To calculate the system limit of detection (sLoD), the resolution was determined as three times the standard deviation of a representative section of baseline during a sensing experiment (3σ), divided by the experimentally determined bulk sensitivity (*S*_exp_).

$$sLoD=\frac{3\times \sigma }{S_{\exp}}$$ 5

## Experimental Section

### PIC Fabrication

The racetrack Si_3_N_4_ ring resonator, designed for quasi-TE mode was fabricated on a silicon substrate with a 2.2 μm buried oxide layer and carrying a 300 nm thick Si_3_N_4_ layer using electron beam lithography patterning and ICP plasma etching. The ring and bus Si_3_N_4_ waveguides thus measured 300 nm in height and were 1.1 μm wide. The bus waveguide extended across the 7.1 mm wide chip with the ring resonator located in the center. The radius, coupling gap and coupling length were 33 μm, 0.4 and 2.75 μm, respectively. The device includes a partial transmitting element (PTE) in the bus waveguide at the straight waveguide section in the coupling region with 0.57 μm periodicity, introducing a controlled coupling imbalance. This results in a slightly asymmetric Fano shape in the MRR spectrum (Figure 2a), which can be used to tune the resonance slope for adjusting linearity and sensitivity in the fixed wavelength read-out modality described in the Experimental Section. Although the asymmetry is modest and does not result in a pronounced Fano resonance, it helps optimizing the extinction ratio and measurable signal amplitude. A SEM image of the fabricated ring resonator and a typical spectrum recorded with water cladding can be seen in Figure 2b. The *Q*-factor of the resonance at 1575 nm used for the sensing experiments was 9.4 × 10^3^.

**Figure 2:**
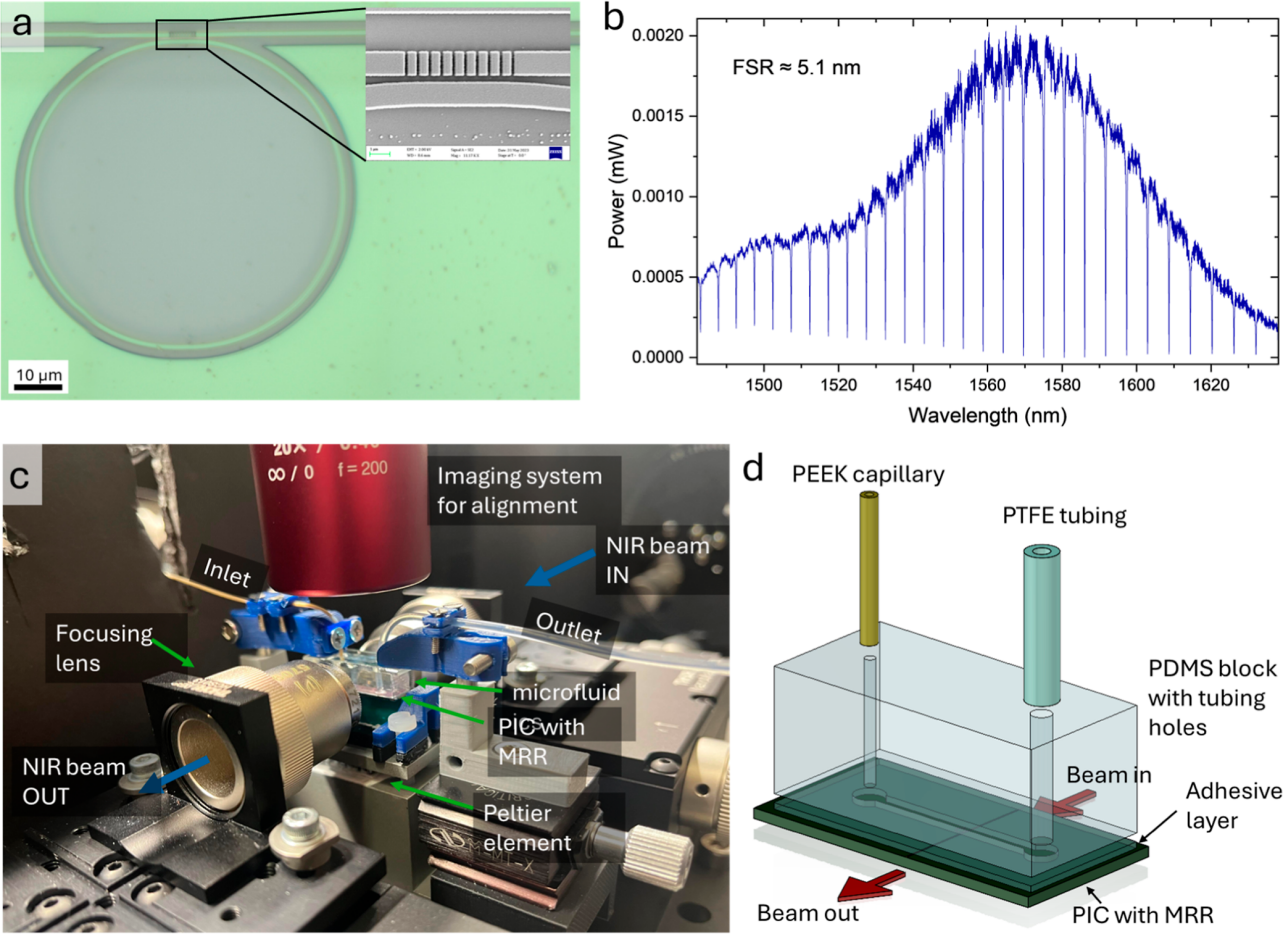
(a) Image of the ring resonator with the inset showing a detailed SEM image of the coupling region, (b) ring resonator spectrum with deionized water as cladding, (c) image of the setup, (d) schematic structure of the microfluidics on top of the PIC (not to scale).

### Microfluidics Fabrication

Custom microfluidics were fabricated from PDMS (Sylgard 184) with a 49 μm thick adhesive layer (Adhesive Research 92712) between the PIC carrying chip and the PDMS block containing holes punched for the in- and out-let fluid connections. The channel structure (length: 11 mm, width: 0.75 mm) was cut into the adhesive foil layer, plasma-activated and then bonded to a plasma-activated PDMS block, making a connection to the inlet and outlet for the fluid. Subsequently, the other side of the adhesive layer was plasma-activated and bonded to the chip, positioning the channel on top of the ring resonator oriented 90° to the orientation of the bus waveguide. This design resulted in a channel height of 49 μm and a width of 0.75 mm in which the ring resonator was placed. Considering the diameter of the ring (33 μm), the effective mode volume and the spatial mode overlap with the sample the actual detection volume can be estimated to be ∼7 fL.^41,42^ When considering the thickness and width of the flow channel and assuming the ring resonator is placed in the middle of the channel, the volume from the inlet to the MRR can be calculated to be 0.2 μL which is still considerably smaller than the internal volume of the commercial RI detector which is stated to be 60 μL. For the inlet, a 1/32″ PEEK capillary with an ID of 125 μm was used to minimize sample dispersion due to axial and radial analyte diffusion in the strongly laminar flow conditions prevailing in the capillary and the microfluidic flow channel. For the outlet a PTFE tubing with 1.5 mm OD and 1 mm ID was chosen to reduce back pressure in the microfluidics.

The chip with the microfluidics was placed on a temperature-controlled copper holder and fixed with a carbon tape. The holder was kept at 24 °C during experiments with fluctuations of the temperature staying below 0.01 K. A glass plate was positioned on top of the microfluidics for greater mechanical stability and specially designed clamps were used to fix the microfluidics in position and to stabilize the in- and out-put tubing. An image of the setup with the microfluidics and a schematic of the microfluidics can be found in Figure 2c,d.

### Experimental Setup

A tunable laser (SanTec TLS 570), operating from 1480 to 1640 nm and connected to a polarization maintaining fiber, was used to couple NIR radiation to the Si_3_N_4_ PIC. The polarization was optimized using a paddle controller (Thorlabs FPC562) and the light was then collimated and focused on the facets of the waveguide via butt coupling. The light was collected again using two lenses and led to the Powermeter (SanTec MPM 210-H) for detection by another polarization maintaining fiber. To characterize the MRR, the full tuning range of the laser was used. For the data acquisition modality termed “sweeping mode” a range of 0.5–4 nm around a chosen resonance was repeatedly scanned, acquiring one spectrum every 1–2 s. The experiment was additionally performed at a fixed “single wavelength mode”. A chopper was positioned after the first collimating lens (see Figure 3) to allow for amplitude modulation and lock in detection (Zurich Instruments MFLI) to reduce noise. The signal from the power meter was demodulated with the reference signal provided by the chopper. In this modality the data acquisition rate was 120 Hz.

**Figure 3:**
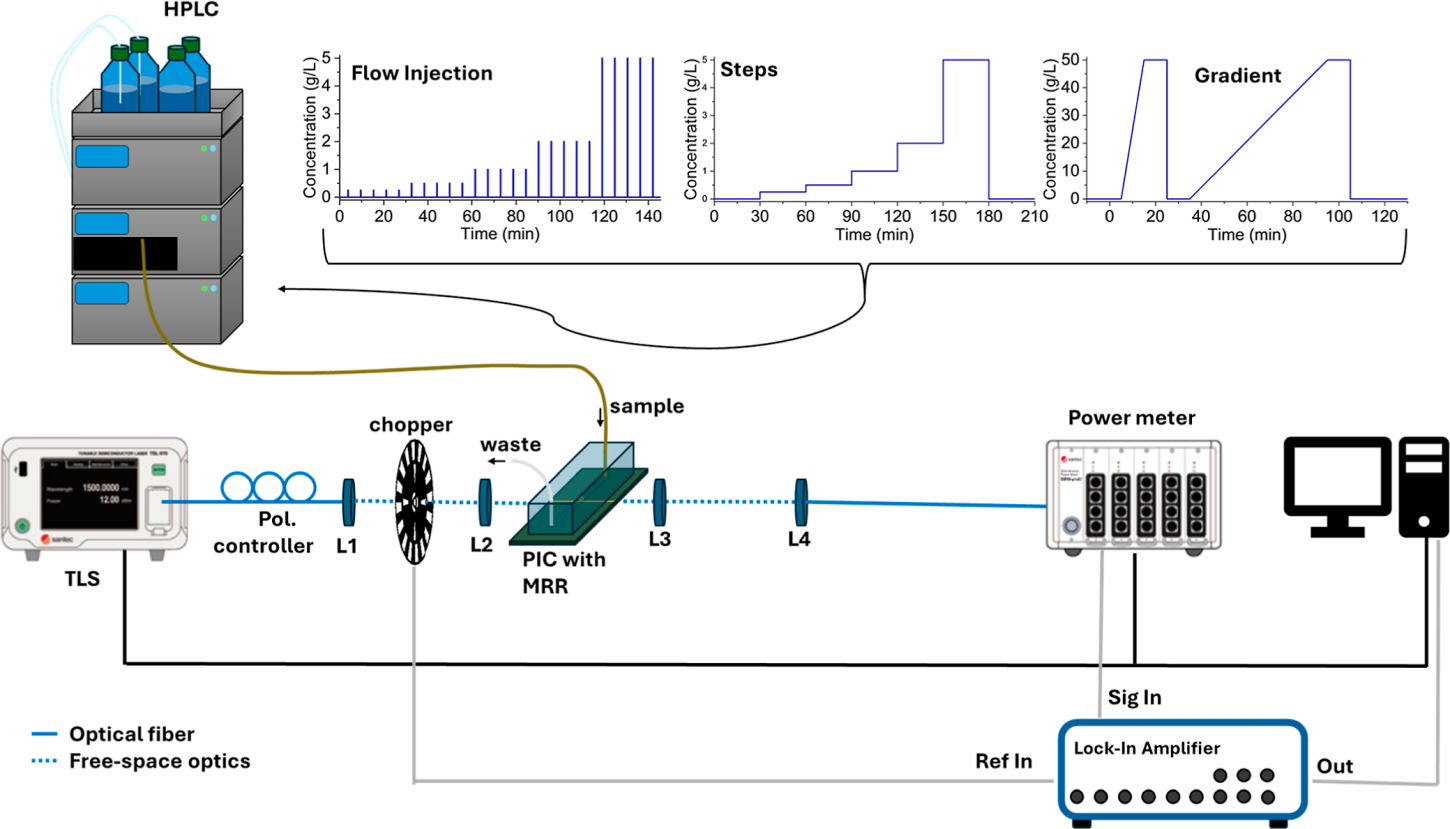
Schematic of the setup used for refractive index sensing experiments in two measurement modalities: sweeping mode, and single wavelength mode. The three different types of sensing experiments for glucose detection are shown at the top.

The setup was encased to further minimize noise, and the input port of the microfluidics was connected to the autosampler of a HPLC system (Dionex, Ultimate3000). A schematic of the setup can be seen in Figure 3.

To provide reference measurements for the flow injection analysis experiment a commercial refractive index detector (Shodex RI-101) was plugged to the flow system replacing the MRR-based sensor. A Sepax, Carbomix Ca column (NP5, 8% cross-linkage, 4.6 × 300 mm) was used for isocratic sugar separation.

## Chemicals

d-Glucose (≥99.5%, Sigma-Aldrich), d-Fructose (≥99%, Merck), d-Sorbitol (≥98%, Sigma-Aldrich) and sucrose (≥99%, Sigma-Aldrich) solutions were prepared in ultrapure water (>18 MΩ, Millipore, Bedford, USA). Water (HPLC-grade) was used for steady solvent supply during experiments.

### Description of Conducted Flow Experiments and Data Acquisition Modes

Three main types of sensing experiments were carried out to evaluate repeatability, dynamic range and long-term stability of the proposed MRR based RI sensor. Figure 3 depicts the experiments carried out which can be categorized as flow injection analysis (FIA) experiments, step experiments and gradient experiments. In each of the experiments, a flow rate of 100 μL/min was employed. For the FIA experiments, the autosampler of the HPLC system was used to inject 10 μL of glucose solutions of different concentrations. For the step and gradient experiment, the mixing function of the HPLC pump was exploited to provide different concentrations from the glucose stock solution and deionized water to the MRR based RI sensor. Prior to each experiment, the temperature was allowed to stabilize for 30 min after closing the enclosure to minimize measurement drift.

Data from each experiment were recorded using the “sweeping” and “single wavelength” measurement modalities. “Sweeping” required data postprocessing, during which the resonance’s wavelength shift (Δλ) could then be extracted from the recorded spectra. In contrast, for the fixed wavelength modality and employing a lock-in amplifier, the laser was first tuned to the steepest inflection point of the target MRR’s resonance at the start of the measurement sequence and kept there for the rest of the experiment. The incoming light to the chip was modulated at 120 Hz. A reference signal from the chopper and the output from the power meter are demodulated by the lock-in amplifier to retrieve the Δ*I*.

The flow injection analysis experiments were also carried out using the commercial refractive index detector (Shodex RI-101) and thus served as a reference for both the sweeping and fixed wavelength data acquisition modality. This was not possible for the step and gradient experiment, due to the limited linear range of the commercial detector. Also, the fixed wavelength measurement modality could not be employed to follow the step and gradient experiments due to its limited linear range as well. For the lock-in method, the linear range can be deduced from the width of the linear section of the resonance dip and the sensitivity.

Finally to demonstrate the applicability of our system as a refractive index sensor in chromatographic conditions using the fixed wavelength modality, an isocratic HPLC separation was achieved using the Carbomix Ca column at 80 °C with a flow rate of 120 μL/min. Twenty μL of a solution containing 4 sugars at 1 g/L each were injected as sample.

## Results and Discussion

To extract the resonance wavelength position for the sweeping measurement modality, the derivative of the spectrum was calculated. A linear fitting was then performed using 30 data points closest to the zero-crossing, and the intersection of the fitted line with the *x*-axis was calculated. The raw data and the extracted magnitude is presented in Figure S1.

Baseline drifts in the lock-in amplifier data recorded from the fixed wavelength modality were removed using an alternating least squares (ALS) algorithm. The raw data of the measurements along with the fitted baseline can be found in Figure S2. Baseline drifts perceived with the sweeping modality were caused by slow temperature changes and were corrected with an exponential function (Figure S3).

Following these preprocessing steps the results for the FIA experiment, using both measurement modalities for the ring resonator and the commercial detector, highlight the distinct traits of each approach. The measurements with the ring resonator have considerably narrower peaks for the injected glucose solutions with FWHM values of 5.3 and 5.6 s for the sweeping and lock-in modality, respectively, as compared to the commercial detector with 9.8 s. The injection peak shape of the ring resonator modalities has a strongly symmetric and Gaussian shape, which is favorable in chromatographic applications.^43^ This can mainly be attributed to the small volume of the microfluidics, leading to negligible broadening of the peaks due to sample dispersion compared to the commercial detector. This is of high importance for applications where low dispersion is needed such as in micro HPLC separations.^44^

The resonance shift of the ring resonator for injected glucose solutions with concentrations from 0.25 to 5 g/L is highly linear, as depicted in Figure 4. For the single wavelength measurement modality the 5 g/L solution exceeded the linear range. For this modality the wavelength span of the linear part of the slope of the target MRR’s spectrum, determines the maximum linear range. Due to the asymmetry of the resonances, the linear range is different for both sides of the resonance. The tunable NIR laser is tuned to the inflection point with the linear part of the slope being 0.09 nm wide at the steeper side of the resonance. Considering the experimental value for the sensitivity in nm/RIU (see below) the linear range should be 4 × 10^–3^ RIU, which aligns well with our experimental findings. It is important to note that the broader linear part of the slope at the other side of the resonance would lead to a higher linear range, at the cost of a lower sensitivity. However, a major advantage of the measurement at a fixed laser wavelength is its fast measurement capability allowing to resolve the shape of the flow injection analysis peak much better than when using the sweeping measurement modality.

**Figure 4:**
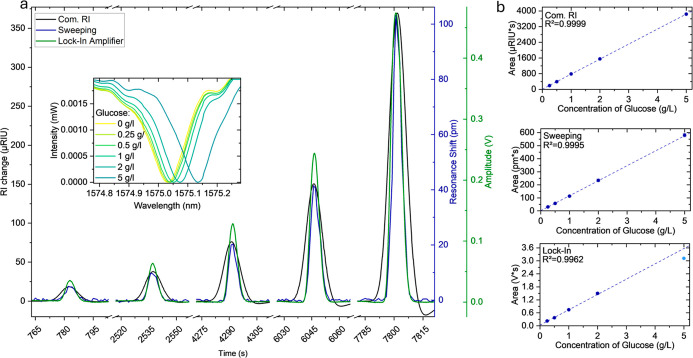
(a) Flow injection analysis peaks of injected glucose samples measured by a commercial detector (black), sweeping the MRR-spectrum (blue) and lock-in detection at fixed wavelength (green). The inset graph shows the maximum shift experienced by the MRR during the injection of each glucose concentration. (b) corresponding regression lines for each measurement type based on the calculated area of the RI peak response (*n* = 5).

For the determination of the sensitivity of the MRR-based RI sensor, glucose concentrations from 0.25 g/L to 5 g/L were rinsed through the channel for 30 min each and the resonance shift was recovered using the sweeping data evaluation modality. The step experiment showed a response of 18.52 pm/(g/L) for glucose. To calculate the sensitivity of the RI-sensor, refractive index data for glucose solutions obtained at 1550 nm by Pereira et al.^45^ stating a change of 1.64 × 10^–4^ RIU/(g/L), were used. The experimental sensitivity of the chosen resonance was 113 nm/RIU which is lower than the theoretically expected 147 nm/RIU. The theoretical bulk sensitivity was calculated following eq 3, using 1.55 μm, 1.58, 2.04, 0.19 for λ_res_, *n*_eff_, *n*_g_ and ∂*n*_eff_/∂*n*_clad_ respectively. The values were obtained using an open-source finite-difference frequency-domain mode solver, which is part of the GDSfactory Python library.^46^ To assess the linearity of the MRR-based RI sensor response over a wider refractive index range, a glucose gradient ranging from 0 to 50 g/L was fed into the flow system, corresponding to a refractive index span of 8.2 × 10^–3^ RIU. The raw data is presented in Figure S4. The MRR-based RI sensor response stayed linear over the entire concentration range, as shown in Figure 5b, which presents a major advantage compared to the commercial refractive index sensor with a limited measurement range of only 5.12 × 10^–4^ RIU.

**Figure 5:**
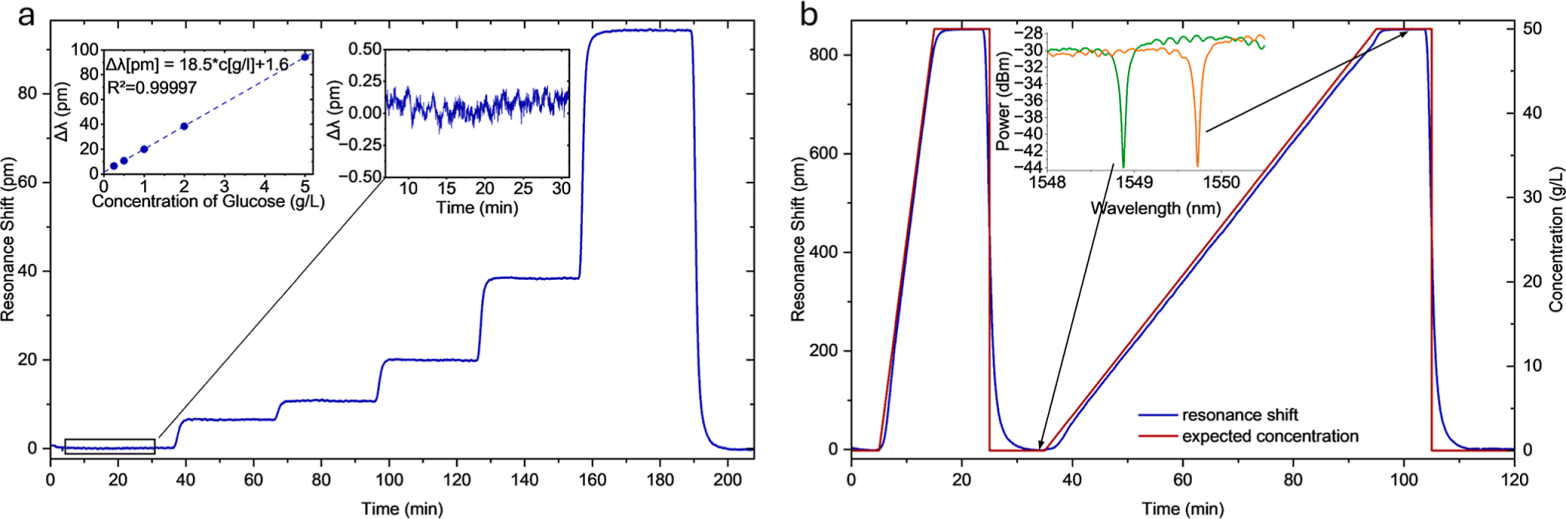
(a) Step experiment with glucose concentrations from 0 to 5 g/L. The left inset graph shows calculated the resonance shift with concentration used to calculate the MRR sensitivity. The right inset shows the noise for a representative section of baseline (b) gradient experiment from 0 to 50 g/L glucose showing shift of ring resonator resonance.

The baseline noise (3σ) for the step experiment was calculated to be 0.26 pm for the section shown in the inset graph in Figure 5a, thus leading to a sLOD of 2.3 × 10^–6^ RIU. This sLOD is among the best reported to date, as seen in Table 1, with comparable sensitivity to other works. While high sensitvitiy plasmonic and photonic-crystal RI sensors have been reported in simulation studies,^47–49^ they generally lack experimental validation and are not compatible with flow-through operation due to metallic losses, complex geometries, or challenging fabrication tolerances. In contrast, our experimentally demonstrated Si_3_N_4_ MRR combines CMOS-compatible fabrication, low optical loss, and enhanced compatibility with microfluidic integration (e.g., non suspended structures), enabling real-time RI detection under realistic chromatographic flow conditions.

**Table 1:** Comparison between Different Experimental Ring Resonator Setups Used for Refractive Index Sensing in Literature^a^ ^,^ ^b^

λ [nm]	coupling method	WG material	experimental sensitivity [nm/RIU]	*Q*-factor	LOD [RIU]	source
1550	EF	Si_3_N_4_	113^b^	9.4 × 10^3^	2.3 × 10^–6^	this work
1550	GC	Si	163^b^	4.3 × 10^4^	7.6 × 10^–7^	^50^
1310	BC in, EF out	Si_3_N_4_	210^b^	5.0 × 10^3^	1.26 × 10^–3^	^12^
1310	GC in, EF out	Si_3_N_4_	240^b^	3.0 × 10^3^	8.8 × 10^–6^	^51^
1550	GC	Si	298^b^	3.3 × 10^2^	4.2 × 10^–5^	^52^
1550	BC	Si_3_N_4_	113	8.0 × 10^4^	7.99 × 10^–4^	^53^
1550	GC	Si	113^b^	-	2.5 × 10^–6^	^16^
1550	GC	Si_3_N_4_	23.1	-	4.46 × 10^–6^	^54^

a End Fire (EF), Grating Coupler (GC), Butt Coupling (BC), waveguide (WG).

b Flow-through experiment.

One important factor in reaching low sLODs for MRR-based RI sensors is minimizing wavelength noise. We found that the wavelength accuracy of the laser system plays an important role in reaching low noise levels. By decreasing the laser scan speed from 100 to 1 nm/s, the baseline noise was reduced from 1.0 to 0.26 pm (see Figure S5), yielding a significantly lower sLOD for the step experiment where a low scan speed was chosen. To determine the sLOD of the single wavelength detection modality, the baseline noise and the signal amplitude at the peaks of the flow injection analysis experiments were compared.

As the different experiment types were carried out over the course of two months, a decrease in sensitivity was noticed, which could possibly be caused by the formation of a thin layer of debris on the surface of the ring resonator. Further research will be conducted to examine cleaning procedures or proper storage conditions for longer periods.

The capability of the proposed sensor system to operate as an RI transducer under chromatographic conditions was validated through the separation of four sugars. Additionally to the baseline removal a low pass FFT filter was applied to the chromatogram, acquired with the fixed wavelength modality removing high frequency noise.

The sensor system successfully resolved sucrose, glucose, fructose, and sorbitol with retention times consistent with those measured by a commercial RI detector (see Figure 6), demonstrating label-free detection under dynamic flow conditions.

**Figure 6:**
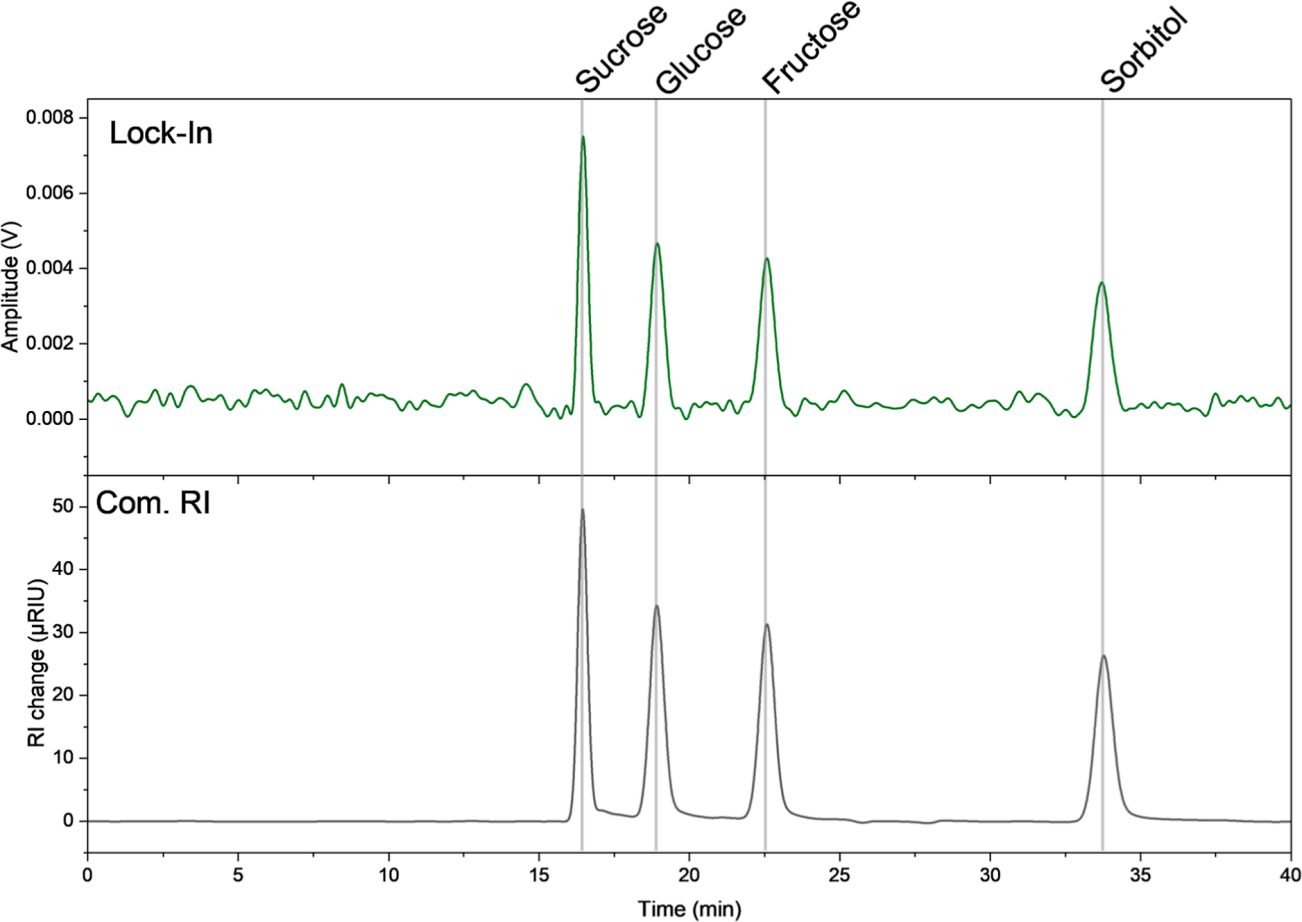
Chromatograms of an isocratic separation of a mixture of sucrose, glucose, fructose and sorbitol (1 g/L each) using the fixed wavelength modality for read-out of the MRR (top) and a commercial RI detector (bottom).

## Conclusion and Outlook

In summary, we have demonstrated a Si_3_N_4_ racetrack-MRR integrated with low-volume microfluidics for flow-through refractive index sensing. Furthermore, implementation and comparison of both interrogation modalities, wavelength sweeping and fixed-wavelength intensity detection, on the same device has been experimentally shown. Depending on the chosen modality, the MRR-based RI sensor can outperform a commercial HPLC detector in terms of peak broadening (i.e., FIA), linear range, and measurement rate, while the sensitivity (sLOD) remains slightly lower. A quantitative comparison is summarized in Table 2.

**Table 2:** Comparison of Our MRR Measurement Setup with a Commercial Refractive Index Detector *Shodex RI-101

	measurement rate [s^–1^]	LOD [RIU]	linear range [RIU]	FWHM (FIA) [s]
sweeping	0.5–1	2.3 × 10^–6^	4 × 10^–3^	5.3
lock-in	>10^2^	2.0 × 10^–6^	<8.2 × 10^–4^	5.6
commercial detector*	0.1–10	2.5 × 10^–9^	5 × 10^–4^	9.8

The sweeping method provides a verified linear range of at least 6.5 × 10^–3^ RIU, exceeding the commercial detector by more than an order of magnitude. Although the free spectral range is sometimes cited as a limiting factor,^55^ shifts larger than the FSR can be resolved if consecutive spectra remain within half an FSR. This broad range comes at the cost of lower measurement speed due to the need for slower, low-noise spectral scans.

Fixed-wavelength interrogation at the resonance slope achieves measurement rates above 10^3^ Hz, over 2 orders of magnitude faster than the commercial detector, while maintaining minimal peak broadening under flow. Based on this analysis, the intensity-based modality was selected to reconstruct chromatograms, enabling label-free detection of four sugars (sucrose, glucose, fructose, and sorbitol) under HPLC conditions and demonstrating the practical analytical relevance of the platform.

Looking ahead, sensor performance in terms of long-term stability can be further enhanced through balanced detection using a reference waveguide or MRR and through improved packaging, such as fiber bonding. These developments will support the evolution of Si_3_N_4_ PIC-based MRR detectors into robust, compact, and high-speed refractive-index sensors for advanced analytical flow systems.

## Supplementary Material



## Notes

The following files are available free of charge. Flow injection analysis raw data sweeping modality, Flow Injection analysis raw data lock-in and fitted baseline, step raw data sweeping modality and fitted baseline, gradient raw data sweeping modality, relationship between laser scan speed and baseline noise (PDF)

## Abbreviations

CMOS: complementary metal–oxide-semiconductor
FIA: flow injection analysis
FWHM: full-width half-maximum
HPLC: high performance liquid chromatography
MRR: microring-resonator
PIC: photonic integrated circuit
RI: refractive index
sLoD: system limit of detection
SOI: silicon-on-insulator
TLS: tunable laser source
