# Supplementary material for: Si3N4 Microring Resonator-Based Refractive Index Sensing for Liquid Samples: Comparing Wavelength Scanning and Fixed-Wavelength Probing
Source: ACS Meas Sci Au. 2025 Dec 15;6(1):116–25. doi: 10.1021/acsmeasuresciau.5c00139 (PMC12921590; doi:10.1021/acsmeasuresciau.5c00139)
Supplement: Supplementary file 1 [file tg5c00139_si_001.pdf]

Supporting Information

Si_3N_4 Microring Resonator-Based Refractive Index Sensing for Liquid Samples: Comparing Wavelength Scanning and Fixed-Wavelength Probing

Daniela Tomasetig,[*,†,∥] *Jesus Hernan Mendoza-Castro,*[†,∥] *Silvia Schobesberger,*[‡] *Artem S. Vorobev,*[§] *Liam O'Faolain,*[§] *Bernhard Lendl,*[*,†]

[†] Institute of Chemical Technologies and Analytics, TU Wien, Getreidemarkt 9/164, Vienna 1060, Austria

[‡] Institute of Applied Synthetic Chemistry, TU Wien, Getreidemarkt 9/163, Vienna 1060, Austria

[§] Centre for Advanced Photonics & Process Analysis, Munster Technological University, Rossa Avenue, T12 P928 Cork, Ireland

Description of contents:

Figure S1: Flow Injection analysis raw data sweeping modality

Figure S2: Flow Injection analysis raw data Lock-In and fitted baseline

Figure S3: Step raw data sweeping modality and fitted baseline

Figure S4: Gradient raw data sweeping modality

Figure S5: Relationship between laser scan speed and baseline noise

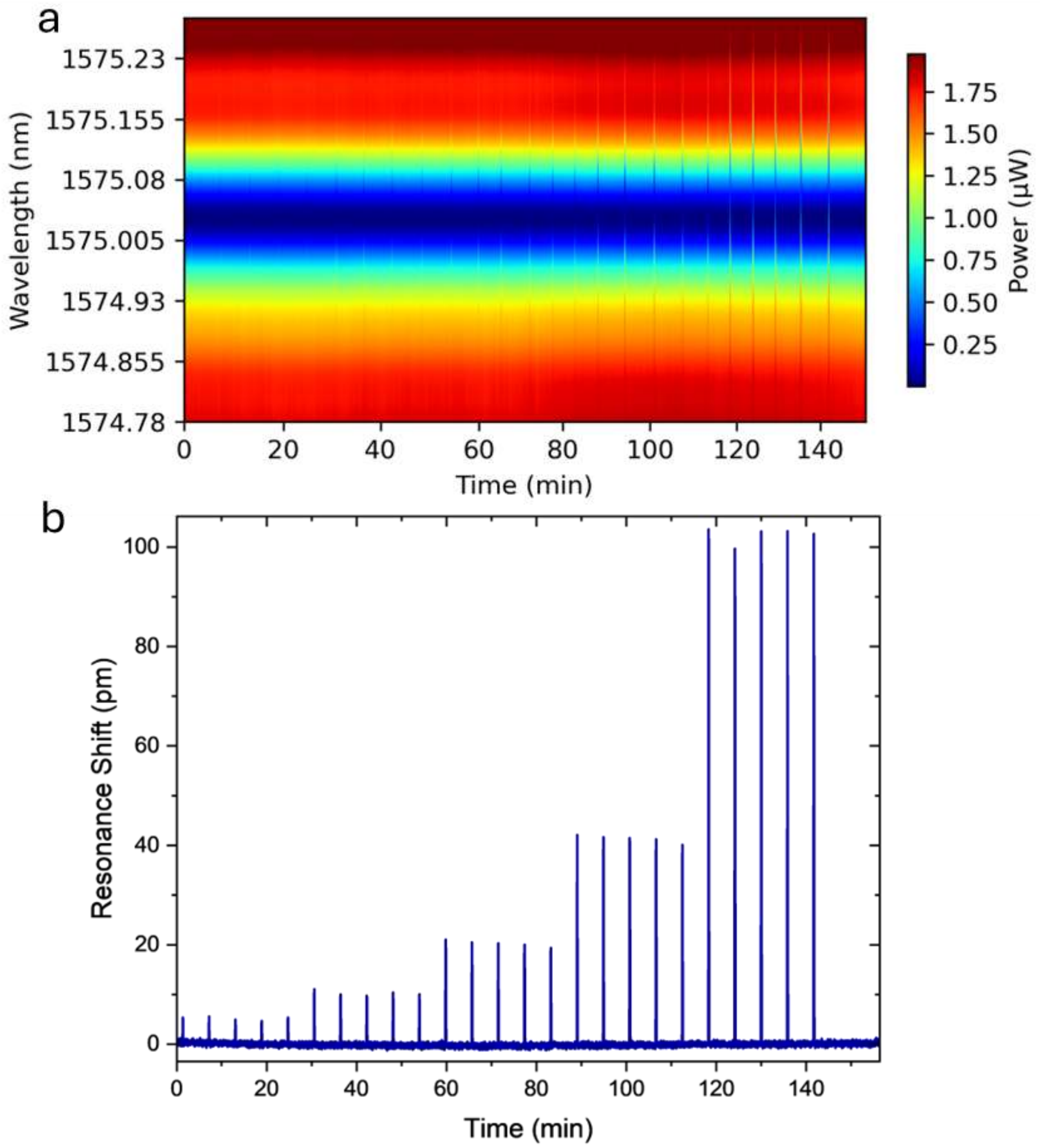

Figure S1: Raw data of the flow injection experiment acquired by sweeping the resonance spectrum. a) acquired spectra over time, b) determined resonance shift

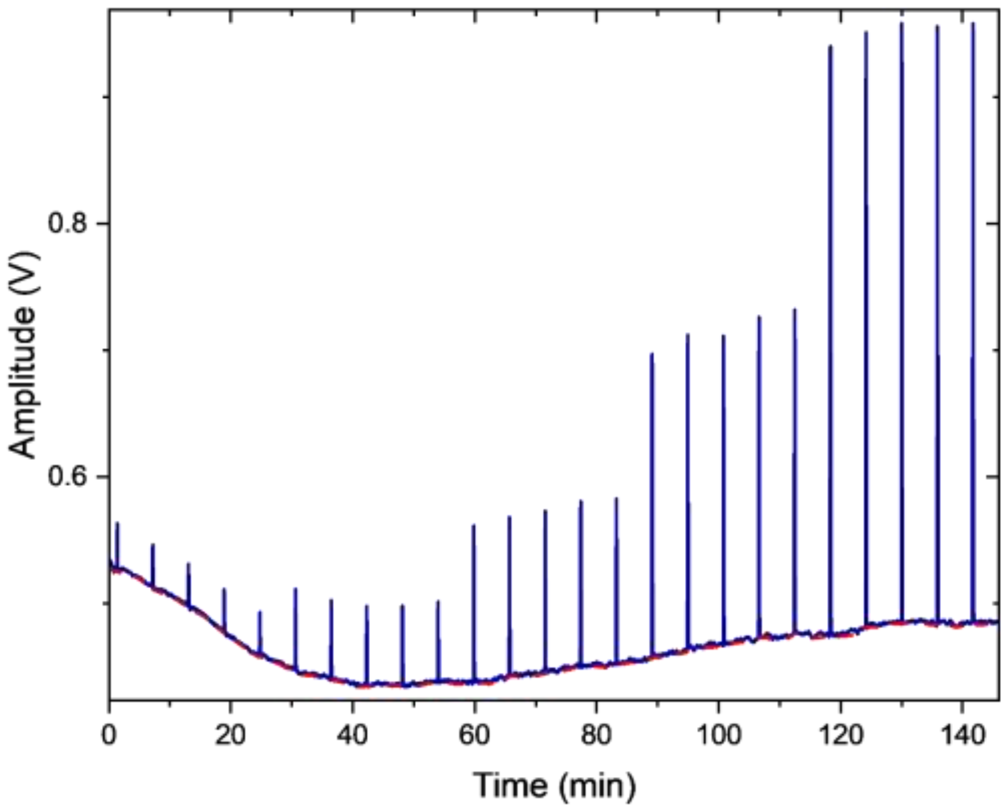

Figure S2: Raw data of the flow injection experiment acquired by Lock-In detection at the slope of the resonance spectrum. The dashed line shows the baseline used for correction calculated by ALS (Asymmetric least squares).

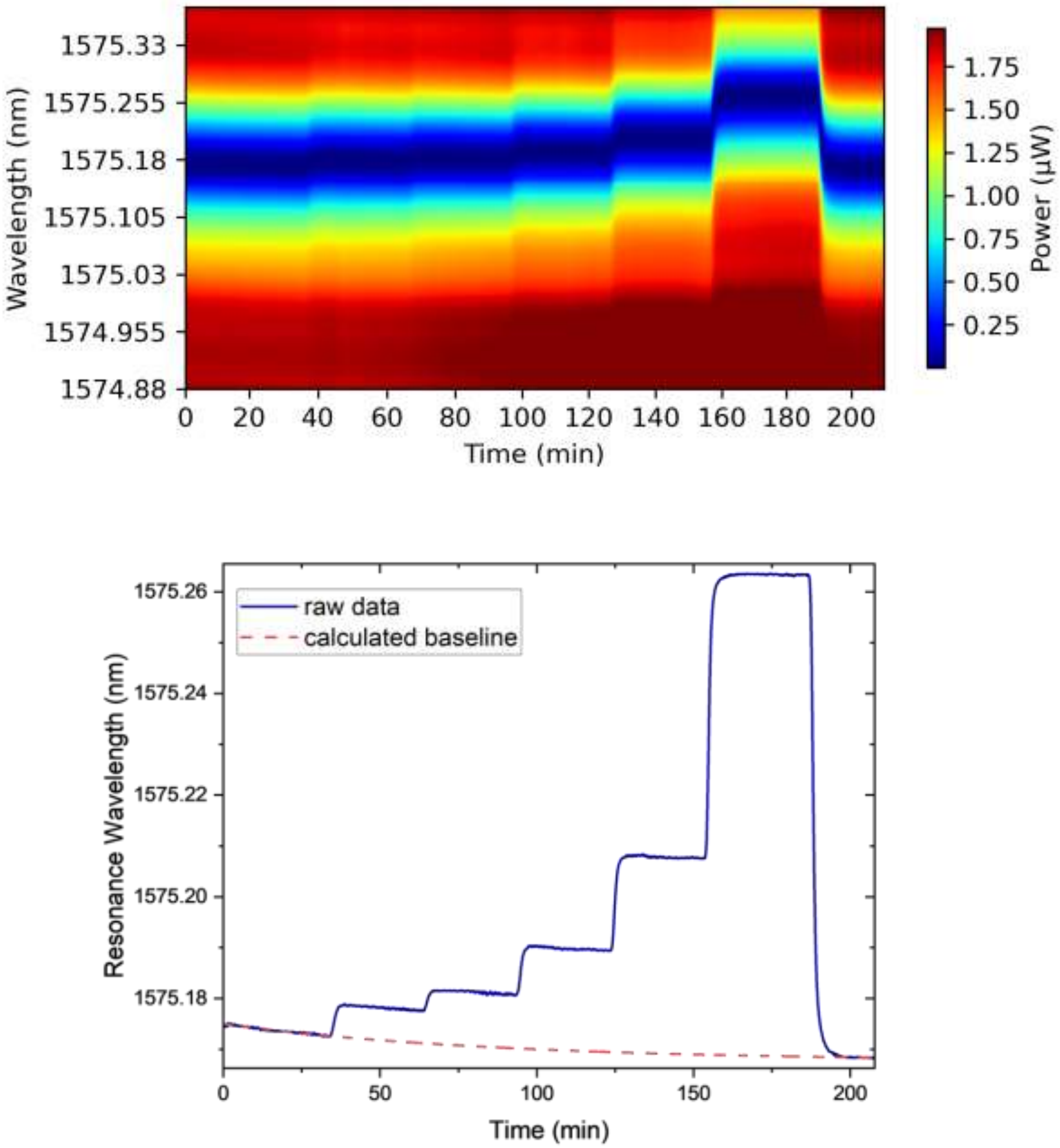

Figure S3: Raw data of the step experiment acquired by sweeping the resonance spectrum. The top figure shows the acquired spectra over time. In the bottom the determined wavelength shift and the exponential baseline used for correction is displayed.

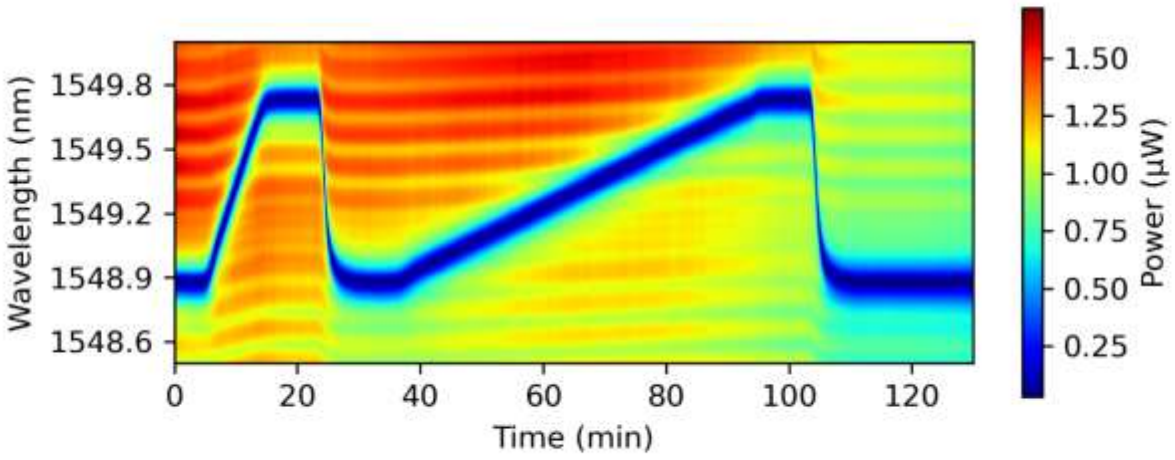

Figure S4: Raw data of the gradient experiment acquired by sweeping the resonance spectrum showing all acquired spectra over time.

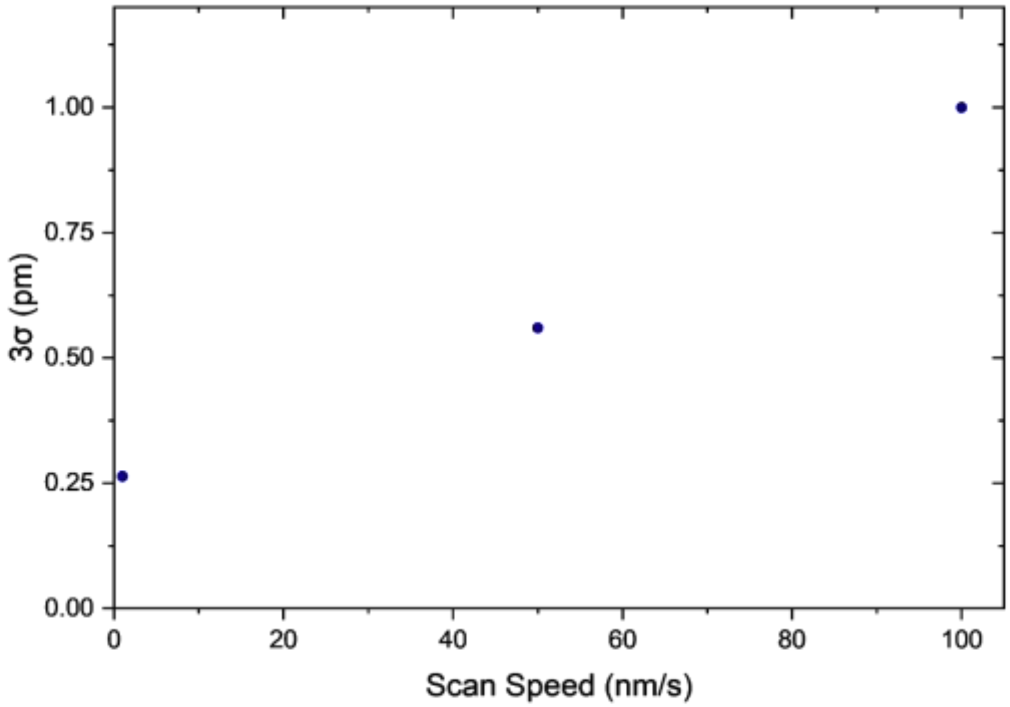

Figure S5: Relationship between the Laser Scan speed and the baseline noise level of the resonance shift
